# Usage of a Value-based Triaging Methodology for Assessing Improvements in Value for Hip Fracture Inpatient Episodes of Care From 2014 to 2019: A Pilot Study

**DOI:** 10.5435/JAAOSGlobal-D-22-00096

**Published:** 2022-10-18

**Authors:** Sanjit R. Konda, Rachel Ranson, Adwin Denasty, Kenneth A. Egol

**Affiliations:** From the NYU Langone Orthopedic Hospital, New York, NY, Jamaica Hospital Medical Center, Queens, NY

## Abstract

The purpose of this study was to demonstrate a novel technology used to measure improvements in quality and value of care for treatment of hip fracture patients.

**Methods:**

A novel value-based triaging methodology uses a risk prediction (risk M) and inpatient cost prediction (risk C) algorithm and has been demonstrated to accurately predict high-risk:high-cost episodes of care. Two hundred twenty-nine hip fracture patients from 2014 to 2016 were used to establish baseline length of stay (LOS) and total inpatient cost for each (16) risk:cost quadrants. Two hundred sixty-five patients between 2017 and 2019 with hip fractures were input into the algorithm, and historical LOS and cost for each patient were calculated. Historical values were compared with actual values to determine whether the value of the inpatient episode of care differed from the 2014 to 16 cohort.

**Results:**

When evaluated without risk or cost stratification, the mean actual LOS and cost of the baseline cohort compared with the 2017 to 2019 cohort were 8.0 vs 7.5 days (*P* = 0.43) and $25,446 vs $29,849 (*P* = 0.15), respectively. This analysis demonstrates that there was only a small change in value of care provided to patients based on LOS/cost over the studied period; however, risk:cost analysis using the novel methodology demonstrated that for select risk:cost quadrants, value of care measured by LOS/cost improved, whereas for others it decreased and for others there was no change.

**Conclusion:**

Risk-cost–adjusted analysis of inpatient episodes of care rendered by a value-based triaging methodology provides a robust method of assessing improvements and/or decreases in value-based care when compared with a historical cohort. This methodology provides the tools to both track hospital interventions designed to improve quality and decrease cost as well as determine whether these interventions are effective in improving value.

Hip fractures are a common affliction in the elderly population. They account for 14% of all fractures yet comprise 72% of overall fracture care costs.^1^ With the rising geriatric population and associated hospitalization costs for hip fracture management, it is imperative to optimize their care.^2^

The Centers for Medicare and Medicaid Services (CMS) is the largest single payer and, in 2015, published a statement aiming to link 85% of Medicare fee-for-service payments to quality or value by 2016 and 90% by 2018. In this same statement, they aimed to have 50% of payments tied to quality or value through alternative payment models by the end of 2018.^3^ Thus, the previous fee-for-service payment models have largely been replaced with alternative payment models.

To replace the fee-for-service model, in 2017, the CMS introduced hip fracture patients into the Comprehensive Joint Replacement and Bundled Payments for Care Improvement bundled payment models.^4^ Because hip fracture cases can be very complex, a broad bundled payment model may not always be the most effective method to guarantee fair reimbursement for participating physicians and hospitals.^5^ Thus, these initiatives by the CMS force orthopaedic surgeons and hospital administrators to reframe hip fracture care through value-based episodes of care.

Value-based care is a reimbursement model that links payment for care delivery to the quality of care provided. Quality of care is related to healthcare outcomes such as mortality, modified by cost^6^; however, the specifics of this definition and how it relates to reimbursement models vary widely. There is an increasing need for well-defined outcomes that can be broadly applicable to each patient's needs^6,7,8,9^ to standardize the definition of value. We propose that risk-stratified patient populations must be identified to predict high-risk and high-cost patients to achieve this goal.

Our group has previously demonstrated the predictive ability of the Score for Trauma Triage in the Geriatric and Middle-Aged (STTGMA) to detect mortality risk in a middle-aged and geriatric trauma cohort. This tool has been validated both within the National Trauma Data Bank (100,000 patients) and prospectively at an urban level 1 trauma center.^10-12^ More recently, our group developed a risk and cost-stratified methodology geared toward identifying areas for physicians and hospitals to improve patient care and lower hospital costs.^13^ With hip fracture care included in bundled payment models, better risk-adjustment methods using granular data and analysis are required to “prevent the creation of financial disincentives” for hospitals caring for more complex cases.^5^ Therefore, the purpose of this study was to demonstrate the methodology by which (1) improvements or deficits in value-based care (defined by length of stay [LOS] [outcome] and inpatient cost) are measured using our novel algorithm and (2) these measurements can be used to conduct “deep dives” into the value of individual episodes of care.

## Methods

From October 1, 2014, to December 31, 2016, 229 consecutive hip fracture patients (OTA 31A-C and periprosthetic hip fractures) aged 55 years and older who underwent surgical treatment at our institution were collected. Each patient was assigned a STTGMA risk and cost score. Their LOS was recorded, and the direct variable cost of their inpatient episode of care was collected. Cost information was obtained from the hospital's cost accounting department. This cohort comprised the baseline data and, therefore, served as the control (baseline) cohort.

Between January 1, 2017, and March 31, 2019, 265 consecutive hip fracture patients meeting the same criteria as mentioned earlier were assigned a STTGMA risk and cost score. Similar to the control cohort, both LOS and direct variable cost of the inpatient episode of care were collected for this experimental cohort. In this study, value was defined as total cost per day (LOS [days]/cost [$]).

The mean age of the baseline cohort was 80 ± 11 years. One hundred forty-eight patients were female (64.6%). The most common mechanism of injury was low-energy (90.8%), and most of the patients ambulated without an assistive device before sustaining a fracture (53.7%) (Table 1).

**Table 1:** Breakdown of Implant Type and Injury, Health, and Functional Status for the 229 Baseline Hip Fracture Patients (2014 to 2016)

Factor	N (%)		N (%)
Injury status			
AIS-head/neck		AIS-extremity	
0	211 (92)	3	224 (98)
1	14 (6)	4	5 (2)
2	0 (0)		
3	2 (1)		
4	2 (1)		
AIS-chest		Glasgow Coma Scale score	
0	212 (92.5)	15	209 (91)
1	14 (6)	14	12 (5)
2	3 (1.5)	13	3 (1.5)
		12	2 (1)
Injury mechanism		11	1 (0.5)
High	21 (9)	5	2 (1)
Low	208 (91)		
Health status			
Charlson Comorbidity Index		Anticoagulation therapy	
0	80 (35)	Yes	76 (33)
1	68 (30)	No	153 (67)
2	41 (18)		
3	23 (10)	Albumin	
4	10 (4)	<2.0	1 (0.5)
5	1 (0.5)	2.0-2.9	16 (6.5)
6	5 (2)	3.0-3.9	136 (60)
8	1 (0.5)	4.0-4.9	76 (33)
ASA			
0	25 (10.9)		
1	0		
2	19 (8.3)		
3	113 (49.3)		
4	68 (29.7)		
Functional status			
Ambulatory status		Assistive device use	
Community	156 (68)	Yes	106 (46)
Household	67 (29)	No	123 (54)
Nonambulatory	6 (3)		
Treatment			
Implant type			
Percutaneous pinning	14 (6)		
Sliding hip screw	25 (11)		
Hemiarthroplasty	48 (21)		
Total arthroplasty	10 (4)		
Short intramedullary nail	99 (43)		
Long intramedullary nail	28 (12)		
Periprosthetic fracture fixation	5 (2)		

AIS = Abbreviated Injury Score, ASA = American Society of Anesthesia

In the experimental cohort, the mean age for the entire cohort was 81 ± 11 years. One hundred seventy-eight patients were female (67%) and 87 were male (33%). The most common mechanism of injury was low-energy (95.1%), and most of the patients ambulated with an assistive device before sustaining a fracture (54.3%) (Table 2).

**Table 2:** Breakdown of Implant Type and Injury, Health, and Functional Status for the 265 Experimental Hip Fracture Patients (2017 to 2019)

Factor	N (%)		N (%)
Injury status			
AIS-head/neck		AIS-extremity	
0	254 (95.8)	3	227 (85.7)
1	10 (3.8)	4	38 (14.3)
2	1 (0.4)		
AIS-chest		Injury mechanism	
0	262 (98.9)	High	13 (4.9)
1	1 (0.4)	Low	252 (95.1)
2	2 (0.7)		
Glasgow Coma Scale score			
15	227 (85.7)		
14	32 (12.0)		
13	5 (1.9)		
12	0 (0)		
11	1 (0.4)		
Health status			
Charlson Comorbidity Index		Anticoagulation therapy	
0	88 (33.2)	Yes	61 (23.0)
1	82 (30.9)	No	204 (77.0)
2	41 (15.5)		
3	25 (9.4)	Albumin	
4	14 (5.3)	<2.0	2 (0.7)
5	5 (1.9)	2.0-2.9	11 (4.2)
6	5 (1.9)	3.0-3.9	103 (38.9)
7	4 (1.5)	4.0-4.9	146 (55.1)
12	1 (0.4)	5.0-5.9	3 (1.1)
ASA			
0	9 (3.4)		
1	0		
2	34 (12.8)		
3	167 (63.0)		
4	55 (20.8)		
Functional status			
Ambulatory status		Assistive device use	
Community	170 (64.1)	Yes	144 (54.3)
Household	89 (33.6)	No	121 (45.7)
Nonambulatory	6 (2.3)		
Treatment			
Implant type			
Percutaneous pinning	22 (8.3)		
Sliding hip screw	6 (2.3)		
Hemiarthroplasty	59 (22.2)		
Total arthroplasty	10 (3.8)		
Short intramedullary nail	118 (44.5)		
Long intramedullary nail	36 (13.6)		
Periprosthetic fracture fixation	14 (5.3)		

AIS = Abbreviated Injury Score, ASA = American Society of Anesthesia

The STTGMA tool was developed to stratify risk of inpatient mortality among patients aged 55 years or older. For this study, this validated tool was modified to stratify mortality risk and cost. STTGMA mortality risk (risk [M]) was calculated using data collected from standardized questions obtained in the emergency department (Table 3). The primary outcome measure of this variable, risk (M), was defined as the probability of inpatient mortality ranging from 0% to 100%. Patients were divided into 4 quadrants based on their mortality risk termed minimal (0.00% to 0.50%), low (0.51% to 2.00%), moderate (2.01% to 6.00%), and high (6.01% to 100.00%) risk. STTGMA cost (risk [C]) was calculated using the same variables as risk (M) (Table 3) but included surgical procedure as an additional variable. Surgical procedures consisted of closed reduction and percutaneous pinning, sliding hip screw, short cephalomedullary nail, long cephalomedullary nail, hemiarthroplasty, total hip arthroplasty, and periprosthetic fracture fixation. The primary outcome measure of this variable was defined as the patient's probability of being in the top 5% of costly patients. Therefore, risk (C) was divided into 4 quadrants termed minimal (0.00% to 0.15%), low (0.16% to 5.00%), moderate (5.01% to 25.00%), and high (25.01% to 100.00%) risk of falling into this top 5% category.

**Table 3:** Variables That Comprise the Risk (M) and Risk (C) Scores^a^

Injury Status	Health Status	Functional Status
Low/high energy	CCI^b^	Ambulatory capacity^b^
GCS^b,c^	Anticoagulation (yes/no)^c^	Use of assistive device^b^
AIS-head/neck^b,c^	Albumin level^c^	Age
AIS-chest^b,c^	American Society of Anesthesia score	
AIS-extremity/pelvis^c^		

CCI = Charlson Comorbidity Index, STTGMA = Score for Trauma Triage in the Geriatric and Middle-Aged

a Cost score also includes implant device.

b Indicates variables used to calculate the low-energy STTGMA score.

c Indicates variables used to calculate the high-energy STTGMA score.

**Figure 1:**
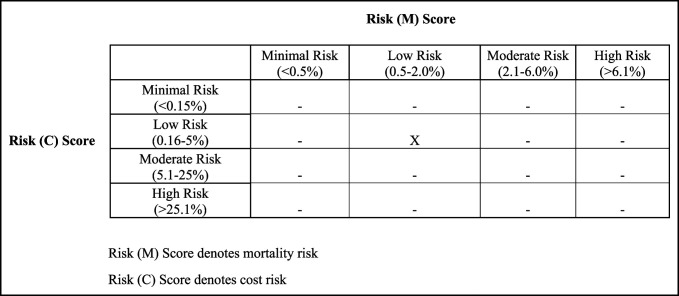
Illustration showing an example of a score grid after inputting patient values into the software. Risk (M) score denotes mortality risk, and risk (C) score denotes cost risk. Both mortality and cost risk scores have four categories: minimal, low, moderate, and high. This figure demonstrates a patient who is projected to be both low mortality risk and low cost risk.

To stratify patients based on both risk (M) and risk (C), a stratification system was designed with risk (M) quadrants along the *x*-axis and risk (C) quadrants along the *y*-axis (Figure 1). This schema was used to assign each patient from the baseline and experimental cohorts into 1 of 16 risk:cost quadrants (Figure 1).^13^ Next, the baseline mean value (previously defined as LOS/cost) of each quadrant was then compared with the corresponding experimental mean value of that respective quadrant to determine whether there was an overall increase, decrease, or no change (equivocal) in the value of episode of care for that specific risk:cost quadrant.

For example, if the mean LOS for an experimental risk:cost quadrant increased from the baseline LOS but the mean experimental inpatient costs for that same case risk:cost quadrant decreased proportionately compared with the baseline costs, this specific risk:cost quadrant value of care was determined to be “equivocal.” Similarly, if both LOS and inpatient costs in the experimental cohort decreased from the baseline cohort, then the value of care for that quadrant would be “improved” and vice versa for increased LOS and costs (Figure 3).

## Results

When evaluated without risk or cost stratification, the mean LOS and cost of the baseline cohort compared with the experimental cohort were 8.0 vs 7.5 days (*P* = 0.43) and $25,446 vs $29,849 (*P* = 0.15), respectively. Using our value methodology of comparing changes in LOS/cost, the experimental cohort had an overall decrease in LOS of 0.5 days but an increase in cost of $4,403. In this scenario, the decrease in LOS was offset by a proportionate increase in total inpatient cost, resulting in no change in value for this inpatient episode of care.

**Figure 2:**
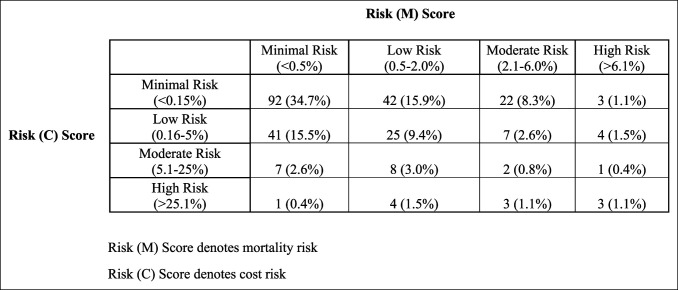
Illustration showing risk (M) score denoting mortality risk and risk (C) score denoting cost risk. Both mortality and cost risk scores have four categories: minimal, low, moderate, and high. This figure demonstrates score assignment distribution for the experimental cohort of 265 consecutive hip fracture patients treated between 2017 and 2019.

**Figure 3:**
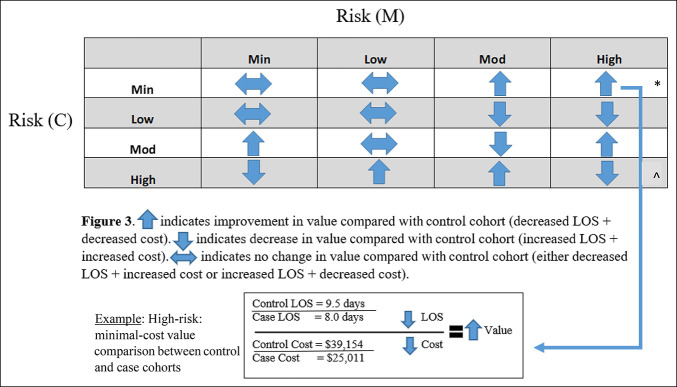
Illustration exhibits value outcomes across each risk and cost-stratified quadrant, defined by baseline versus experimental length of stay (LOS) over baseline versus experimental cost. Note that the baseline cohort is 2014 to 2016 and the case cohort is 2017 to 2019. 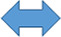 indicates no change in value for the experimental cohort compared with the baseline cohort. This occurs when the baseline cohort has either decreased cost and increased LOS or decreased LOS and increased cost compared with the experimental cohort. 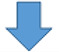 indicates an improved value of care of the case cohort from the baseline cohort with decreased LOS and decreased cost. Alternatively, 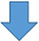 indicates a decreased value of care of the experimental cohort compared with the baseline cohort because of increased LOS and increased costs. The high mortality risk and minimal cost risk group was used as an example to illustrate how value is calculated. The average baseline cohort's LOS was 9.5 days, whereas the experimental cohort's LOS was 8 days, demonstrating decreased LOS. The average cost of the baseline cohort's hospitalization was $39,154, whereas the experimental cohort's hospitalization cost was $25,011, demonstrating decreased costs. The decreased LOS over the decreased cost provided an increased value of care for this risk-stratified group of patients.

When patients were divided into 1 of 16 risk-stratified quadrants, the minimal-risk:minimal-cost quadrant comprised the greatest number of patients (Figure 2). Six quadrants demonstrated an improved value of care, five stayed equivocal, and four revealed a decreased value of care (Figure 3).

Additional granular “drill-down” analyses of the individual patients comprising both the high-risk:minimal-cost and high-risk:high-cost quadrants were conducted (Table 4). In the high-risk:high-cost quadrant, the overall experimental cohort value was decreased (because of increased LOS and increased total costs) compared with the baseline cohort value. When each patient in the experimental cohort was viewed individually, it was revealed that one patient was the primary driver for this poor result (patient 3, LOS 53 days and cost $290,490.35, Table 4), whereas the other two patients had a net increase in value. Comparatively, in the high-risk:minimal-cost quadrant, the overall experimental cohort value was improved compared with the baseline cohort value. Individualized “drill-down” analyses revealed that two patients in the experimental cohort had improvements in value and one patient had no change in their value.

**Table 4:** Individual Case Outcome Breakdown Across *High-Risk:Low-Cost and ^High-Risk:High-Cost Quadrants

Quadrant	*High-Risk:Minimal-Cost Patient 1	*High-Risk:Minimal-Cost Patient 2	*High-Risk:Minimal-Cost Patient 3	^High-Risk:High-Cost Patient 1	^High-Risk:High-Cost Patient 2	^High-Risk:High-Cost Patient 3
STTGMA risk score (%)	7.9	12.1	13	95.1	8.5	35.8
STTGMA cost score (%)	0.1	0.0	0	99.9	98.6	96.8
Baseline mean quadrant cost (US dollars)	39,154	39,154	39,154	84,731	84,731	84,731
Baseline mean quadrant LOS (d)	9.5	9.5	9.5	20	20	20
Experimental mean quadrant cost (US dollars)	25,012	25,012	25,012	122,476	122,476	122,476
Experimental mean quadrant LOS (d)	8	8	8	23.7	23.7	23.7
Experimental individual cost (US dollars)	22,665.91	27,427.15	24,942.55	36,975.23	39,962.52	290,490.35
Experimental individual LOS (d)	5	10	9	10	8	53
Experimental individual cost value	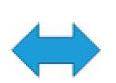	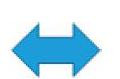	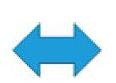	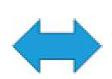	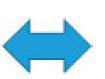	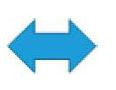
Experimental individual LOS value	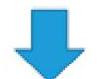	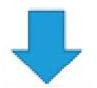	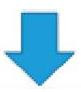	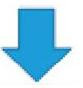	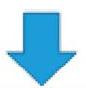	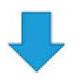
Experimental individual overall value	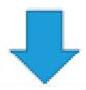	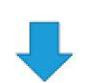	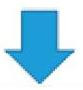	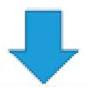	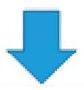	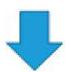
Experimental quadrant overall value	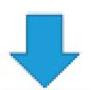	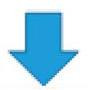	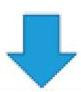	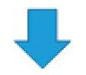	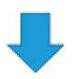	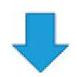

LOS = length of stay, STTGMA = Score for Trauma Triage in the Geriatric and Middle-Aged

Note that * and ^ correspond to the *high-risk:low-cost and ^high-risk:high-cost quadrants from Figure 3. Baseline refers to the 2014 to 2016 cohort and Experimental refers to the 2017 to 2019 cohort.

## Discussion

The STTGMA risk:cost value analysis is an effective methodology to assess the efficacy of hospital quality metric changes in hip fracture care. The subdivision of hip fracture patients into risk and cost-stratified quadrants is advantageous to thoroughly evaluate the efficacy of hospital protocol changes and physician efficiency. To reiterate, when evaluated without risk or cost stratification, the mean actual LOS and cost of the baseline (historical) cohort compared with the experimental (2017 to 2019) cohort were 8.0 vs 7.5 days (*P* = 0.43) and $25,446 vs $29,849 (*P* = 0.15), respectively. The LOS decreased but was offset by an increase in cost. This analysis suggests that there was no gross change in value of care provided to patients based on LOS/cost over the studied period; however, risk:cost analysis yields a more precise analysis of value of care for each patient and is able to identify the patient populations that need additional improvements (ie, outlier patients).

When additional subanalysis of the stratified quadrants was conducted, we were able to assess the individual patient value outcome and its subsequent effect on the value outcome of the overall quadrant. This detailed tracking and analysis is useful for physicians and hospital administration to elucidate deficits and areas for improvement.

Using this methodology, care pathways can be established to optimize the value of each inpatient episode of care. For example, the high-risk:high-cost quadrant demonstrated a decreased value of care from previous years. Now that this quadrant has been identified as weak, it can be a target for physicians and hospital administrators to focus quality and care improvements. Going forward, if a hip fracture patient is triaged into this quadrant, the patient can be more closely followed during their hospitalization to ensure that the value of the inpatient episode is optimized.

Risk-adjusted patient outcome models are becoming more necessary in modern medical care with the increasing trend toward bundled payment reimbursements.^3,7^ The lack of a common risk-adjustment model creates difficulties for establishing national registries to report outcomes and, therefore, inhibits adequate negotiation with payers for value-based payment incentives.^9^ There are currently larger entities that use value-based care algorithms packaged into software for use at the macro level, one of which being a software created by 3M. 3M aggregates data, compares populations, and calculates total cost of care for payers.^14^ Typically, these types of software are used on the back end by hospital administrators for quality control and neither able to be used in real time nor accessible to providers to guide decision making.

A limiting factor of this study is the definition of value. LOS was chosen arbitrarily as a marker for hospital quality outcomes. In reality, there are a host of quality outcomes that could be used to form a more robust definition of quality outcomes. These include but are not limited to incidence of major or minor complications (or hospital acquired conditions), mortality, patient-reported outcome measures such as pain scale and Patient-Reported Outcome Measurement Information System scores, discharge disposition, and readmission rates. In future iterations of this algorithm, there is the capacity to include these factors into the quality outcome aspect of the equation. In addition, more accurate parameters could be set to determine the change in value compared with the control cohort. For example, although inpatient cost may increase, there could be an overall increase in value if the quality outcome measures improved above a predefined cost threshold that was deemed acceptable to achieve those quality outcome improvements. These are just some of the considerations that will be made as this novel pilot risk-cost value algorithm continues to undergo refinement.
